# IoT Enabled Intelligent Stick for Visually Impaired People for Obstacle Recognition

**DOI:** 10.3390/s22228914

**Published:** 2022-11-18

**Authors:** Muhammad Siddique Farooq, Imran Shafi, Harris Khan, Isabel De La Torre Díez, Jose Breñosa, Julio César Martínez Espinosa, Imran Ashraf

**Affiliations:** 1 National Centre for Robotics and Automation, National University of Sciences and Technology (NUST), Islamabad 44000, Pakistan; 2 College of Electrical and Mechanical Engineering, National University of Sciences and Technology (NUST), Islamabad 44000, Pakistan; 3 Department of Signal Theory and Communications and Telematic Engineering, University of Valladolid, Paseo de Belén 15, 47011 Valladolid, Spain; 4 Higher Polytechnic School, Universidad Europea del Atlántico, Isabel Torres 21, 39011 Santander, Spain; 5 Department of Project Management, Universidad Internacional Iberoamericana, Arecibo, PR 00613, USA; 6 Universidade Internacional do Cuanza, Cuito, Bié, Angola; 7 Department of Project Management, Universidad Internacional Iberoamericana, Campeche 24560, Mexico; 8 Fundación Universitaria Internacional de Colombia, Bogotá 111311, Colombia; 9 Department of Information and Communication Engineering, Yeungnam University, Gyeongsan 38541, Republic of Korea

**Keywords:** smart stick, biomedical device, Internet of Things, obstacle detection

## Abstract

This paper presents the design, development, and testing of an IoT-enabled smart stick for visually impaired people to navigate the outside environment with the ability to detect and warn about obstacles. The proposed design employs ultrasonic sensors for obstacle detection, a water sensor for sensing the puddles and wet surfaces in the user’s path, and a high-definition video camera integrated with object recognition. Furthermore, the user is signaled about various hindrances and objects using voice feedback through earphones after accurately detecting and identifying objects. The proposed smart stick has two modes; one uses ultrasonic sensors for detection and feedback through vibration motors to inform about the direction of the obstacle, and the second mode is the detection and recognition of obstacles and providing voice feedback. The proposed system allows for switching between the two modes depending on the environment and personal preference. Moreover, the latitude/longitude values of the user are captured and uploaded to the IoT platform for effective tracking via global positioning system (GPS)/global system for mobile communication (GSM) modules, which enable the live location of the user/stick to be monitored on the IoT dashboard. A panic button is also provided for emergency assistance by generating a request signal in the form of an SMS containing a Google maps link generated with latitude and longitude coordinates and sent through an IoT-enabled environment. The smart stick has been designed to be lightweight, waterproof, size adjustable, and has long battery life. The overall design ensures energy efficiency, portability, stability, ease of access, and robust features.

## 1. Introduction

According to the famous philosopher and scientist Aristotle, our knowledge about the outside world relies upon five sensory organs, and sight is one of those five organs. People who are totally blind or somewhat suffering from visual perception issues fall in the category of visually-impaired people. There are around 2.2 billion people, at least, that have a near or far vision impairment [1]. Children with premature severe vision impairment may experience a lasting impact on their motor, verbal, and emotional abilities, etc. Similarly, they may have poor academic performance. The standard of living is significantly impacted by vision impairment in adults. Adults with visual impairment frequently have decreased workforce participation and productivity levels as well as greater rates of anxiety or depression. Older people can be prone to a higher risk of falling due to vision impairment. Similarly, they may feel difficulty in walking and doing daily tasks, and are socially isolated, which may lead to early admission into nursing or care facilities.

The term `visual impairment’ (VI) is used to describe reduced eyesight that cannot be corrected by surgery, medication, or refractive lenses or glasses. Because of this, it leads to cognitive visual system abnormalities that may include permanent vision loss, a small visual field, diminished contrast sensitivity, increasing susceptibility to glare, and a decreased capacity for daily activities such as reading and writing. People bearing a horizontal vision field of less than or equal to 20 degrees with both eyes open (normal vision field is 180 degrees horizontally) or having a visual acuity of 6/60 according to the Snellen chart, are termed as blind. Different disabilities are out there, limiting people from even doing daily tasks that vary from person to person, e.g.,

- Vision loss and blindness,
- Physical disability,
- Speech and language disorders,
- Hearing loss and deafness, etc.

Compared to other disabilities, visually impaired people are the people that encounter the most risks and difficulties. Among all senses, eyesight is one of the most important senses; about 80 percent of what we feel or perceive comes through the sense of sight. When any other sense, such as hearing, smell, etc., stops working, it is the eyes that best protect us from danger. Hence for people who are suffering from visual perception, their vision needs to be substituted by auditory sense or tactiles. According to the National Eyes Survey (NESII) conducted in Malaysia [2], the major cause of blindness was a delay in cataract surgery which caused 216,000 blind and 272,000 to be visually impaired. The second most common cause of blindness was diabetic eye disease, which results in 10 percent being blind and 6 percent with low vision. Therefore, increasing the number of people with visual disabilities attracts the attention of researchers to develop different innovations in the field of assistive technology, hoping that these innovations can help visually disabled individuals in completing their tasks in regular day-to-day existence, the same as ordinary people.

Traditionally visually impaired people used to walk with a stick made up of wood, but as time passed, that got replaced by the aluminum cane. However, as the advancement of technology continues and we go into the modern age and an era of automation, this walking blind stick can no longer provide independence for the visually impaired. As should be clear, numerous assistive technologies have been created over the years, including the smart stick itself, the Mowat sensor, SensCap, Laser Cane, Embedded Glove, and Nav Belt [3]. The smart stick was the most common form of early assistive technology. The goal of assistive devices is to reduce accidents involving visually impaired people in whatever proportion is conceivable. The NAV Belt is used at the waist, and similarly, Sens Cap should be placed on the head of the user, which can cause damage to the neural system. Therefore, such devices are not suitable for the visually impaired. Consequently, smart sticks emerged as a solution for the visually impaired to go out in the world. The smart stick is equipped with different sensors through which it detects obstacles and alerts the user with different methods, e.g., through beep, vibration, and also through voice alerts.

Despite the availability of several existing solutions, they suffer from exact location finding, ease of use, emergency response facilities, etc. The proposed design for the smart stick is the Internet of Things (IoT), which enables and employs several available sensors including the ultrasound, water sensor, and GPS/GSM sensor. It has two modes where an ultrasound sensor is utilized to detect the obstacle and inform the user about the direction of the obstacle via vibration motors. The second module, on the other hand, performs the detection and recognition of the obstacles and provides voice feedback. These modes are switchable as per the priority of the user. A water sensor is employed to detect water puddles or wet surfaces to avoid falling. Moreover, GPS/GSM is used to acquire the exact location of the user with latitude and longitude. Last but not least is the panic button on the smart stick, which can be pressed by the user to obtain an emergency response in case of urgency. The novelty of the proposed smart stick includes the custom design of an adjustable stick for visually impaired people of different heights, and waterproof sensors and a waterproof control box have been used, making the smart stick robust to harsh environments. A custom water sensor has also been designed with smart placement for accurate detection of the puddles; vibration motors to guide the user to navigate around the obstacle and earphones to play the relevant audio message for alerting about the direction of the obstacle for ease of use for the user. The sensors have been placed so as to not alter the holding style of the traditional cane for the user’s ease of use. The learning curve for the proposed design is almost negligible as compared to the ones already found in the literature.

The rest of this paper is organized as follows. The literature review is presented in Section 2, where several works on assistive technology for the visually impaired are discussed. Section 3 provides the details of the proposed smart stick and its working functionality. Results and discussions are given in Section 4 and Section 5, respectively, while the study is concluded in Section 6.

## 2. Literature Review

Visually-impaired people face many difficulties regarding their routine work. They face difficulties while navigating, and they are not able to move freely in an environment known or unknown because of the lack of sight. They need support to know if they are going in the right direction or not, whether they are going to hit obstacles, or if there is water in the way, etc. Obstacles can be identified as a benefit of the rapid technological advancements in both hardware and software. It is challenging for visually impaired people to walk comfortably and without any fear without human help in urban or ambiguous environments. To travel independently, they usually use a cane or a guide dog. The existence of smart sticks should be able to help the visually impaired person navigate the city through their day-by-day movement. The utilization of smart sticks ought to be advanced to be usable and moderate enough that it can be effectively used by all the visually impaired. Along these lines, utilizing the smart stick, which will navigate the visually impaired more precisely, would require the utilization of ultrasonic, passive infrared (PIR) sensors and vibration motors, and a speaker with voice messages saved joined all together to form a model. In this regard, several approaches and systems have been presented in the literature.

The smart stick, developed by [4], uses an ultrasonic sensor for the detection of the objects or obstacles that come in front of the user. The study uses sound waves having a frequency greater than 20 kHz generated through a transmitter of ultrasonic sensors, and the distance is measured through the echo received by the receiver. For the distance of an object from the user sensor, the time interval between transmitting high-frequency sound waves and the receiver was calculated. Ultrasonic sensors can consider a surface with any shape, and it is not influenced by physical contact [5]. It is demonstrated by [6] that the distance and angle estimations of ultrasonic are accurate enough and the relative errors and variances of the estimations are inside a small range.

**Figure 1:**
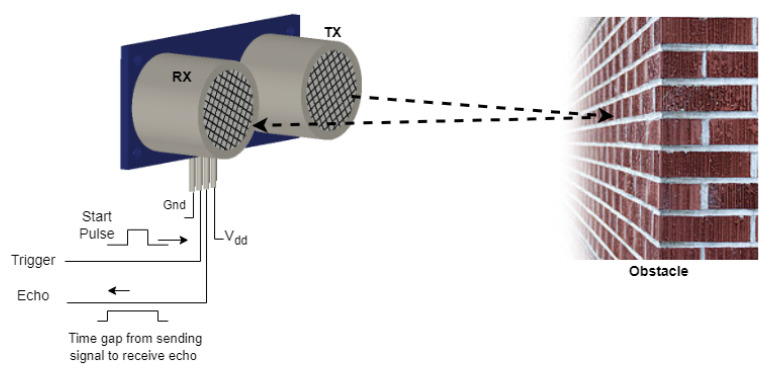
Working of the ultrasonic sensor.

A water sensor is intended to determine the presence of water when the smart stick is dipped into it. By utilizing the water sensor, when it comes in contact with water, it will short the circuit resulting in a closed circuit providing the desired output. The water sensor is valuable in a typically involved region close to any apparatus that can spill water [7]. A motor with unbalanced weight is a vibration motor that alerts the user of the obstacles coming their way through haptic feedback. According to [8], for the detection of an obstacle, two different categories of sensors can be used, namely active sensors and passive sensors. The main difference between active and passive sensors is that active sensors send a signal and then receive a distorted signal when it is reflected. Whereas in the case of a passive sensor, it only receives a signal, i.e., it detects reflected or transmitted electromagnetic signals provided by a natural energy source. To determine the distance between the user and the obstacle, four different sensors can be used: laser sensor, ultrasonic sensor, infrared (IR) sensor, and radar sensor (Figure 1).

Most of the research involves the use of the ultrasonic sensor for the detection of obstacles by emitting and receiving reflected waves from the object. Later, a vibration, beep, or an audio message stored in the memory is used according to the obstacle distance. Some advanced smart sticks utilize GPS in combination with the main system. Additionally, it is important to know that the GPS receiver is helpful to determine the current location of visually impaired people. Such a system has been implemented in the smart sticks by [6,9,10,11]. However, they can not identify obstacles such as descending stairs, gaps, etc. Another problem is that these smart sticks generally only communicate through vibrations and sound beeps, and over time it becomes annoying for the user. In addition, the fact that the user has to remember all the combinations of vibrations and beeps for different obstacles is also difficult. To overcome these problems, [8] developed the effective fast response smart stick for blind people. This system was capable of playing unique audio messages through a speaker or earphone and vibrating after the detection of stairs, puddles, or an object. They accomplished an exceptionally quick reaction time determined as 39 ms in normal distance ≤400 cm before hitting the obstructions.

According to Nada et al. [12], ultrasonic sensors have large sizes, add a lot of weight, and require a large amount of power to operate. They are restricted because of numerous reflections, whereas the laser sensors have an extremely narrow spectrum and are sensitive to ambient light. Thus, it gathers data about the smallest area in the front resulting in poor results. The width of the IR sensor beam lies between laser and ultrasonic, and it has a low power utilization and minimal cost contrasted with laser and ultrasonic sensors. The smart stick proposed in [12] is lightweight and cost-effective, and has a price not exceeding 120 dollars, is foldable, and the obstacle detection alerts through unique voice messages and stairs detection whether they are going upward or downward. A cost-effective yet less useful smart stick for the visually impaired was introduced by [13]. It consisted of an ultrasonic sensor for obstacle detection with a very small range and providing feedback through a buzzer and a light sensor for detecting the ambient light, as well as turning on a light emitting diode (LED) to let the people know of the user’s presence. The design was too simple for its own good. The use of multiple ultrasonic sensors would have added to the applicability of the device while still maintaining its cost-effective factor. The aluminum material for the cane was chosen wisely due to it being lightweight and having a low price. A similar low-cost and ultimately low-on-features smart stick was designed by [14], except for the use of the PIC microcontroller 16F877A and providing a range of 35 cm only.

The study [15] showcased the smart stick and formally verified it using UPPAAL software. The design consisted of two ultrasonic sensors at the top and bottom for obstacle detection and an IR sensor for staircase detection. Due to the low range or IR signal and limitations due to ambient light, this is a relatively good option to detect staircases right in front, as the visually impaired face problems with staircases as well. This would, however, detect other objects at the same distance and height, and the mentioned IR sensor has a range of nearly 1–2 cm. This would not be ideal for detection. The main highlight of the paper, however, was an SD card module with voice messages stored for playing through a speaker when the stairs, water, or obstacle is detected, each having its own audio file. A functional and thoroughly well-thought design of the smart stick for the visually impaired is developed that detects the obstacles below and above the knee, and for this purpose, three ultrasonic sensors were utilized effectively [16]. One ultrasonic was placed in front for the front obstacle detection; the other two were used for above and below-knee obstacle detection. A moisture sensor for the detection of puddles provided feedback using the buzzers to alert the user. The novelty of their design consisted of a radio frequency module with a transmitter on the user and a receiver on the stick for finding the stick if it is misplaced. The user can press the button on the receiver to send a signal and find the location using the buzzer and vibration motors. Furthermore, the study made comparisons of different available sensors for obstacle detection, which help in selecting ultrasonic sensors for obstacle detection. All of the processing, however, was done on Arduino, which is not capable of efficiently working with multiple sensors at the same time. Similarly, the authors did not use any GPS/GSM modules to send the coordinates of the user to the caretaker in case of an emergency.

Kunta et al. [11] had a rather interesting approach to the smart stick for the visually impaired. They implemented the smart stick by combining the other common methods in the literature and added an additional functionality of the GPS and GSM modules for sending a message to the caretaker of the user on their mobile phone by getting coordinates from the GPS module and sending them through the GSM module. A simple user interface was also developed to allow the user’s caretakers to change the mobile number for the message. The work of [10] is identical to [11] but with the exception of the use of Raspberry Pi instead of Arduino and the removal of the RF module. Raspberry Pi is much more suitable for the use of multiple sensors. [5] excluded the GPS/GSM module, and the rest of the work is similar, but the detection range was lowered to 200 cm from 450 cm. [9] had the same work methodology, but the exception is the use of low-power components and use of MSP430 microcontroller known for low power. They also gave careful thought to battery selection and power optimization through which a 12 h battery timing was obtained. Multiple solutions to helping the visually impaired are proposed by Jegadeesan et al. [17]. They proposed a smart stick, as discussed in the previous literature, along with an alternate glove detection design, but it was discarded due to the necessity of always wearing gloves. Another design of obstacle detection shirts was also discarded due to the user having to wear one type of shirt all the time. The mechanism of the smart stick chosen in the end was similar to those discussed previously, except for greater weight than those discussed earlier. They, however, proposed a local city RFID bus route tracking for the visually impaired, where multiple RFID tags would be installed on the bus stops, and the route is instated to the user through voice feedback. This work, however, was most likely inspired by research conducted in 2005, namely ‘RoboCart: toward robot-assisted navigation of grocery stores by the visually impaired’ by [18]; the assistance is provided solely inside the store via RFID tags that are positioned at various points.

Although substantial work has been conducted on smart sticks; IoT-enabled smart sticks with image processing are far between. Similarly, the physical design of the smart stick has not been given much attention for the comfort of the user, and the implementation of a waterproof smart stick has not been found in the literature. Image processing for recognition of objects in smart sticks is far-fetched; and different combinations of buzzers and vibration motors are used to alert the user of obstacles, which are hard to remember and lack ease of use. This study presents a novel IoT-enabled smart stick for the visually impaired to overcome these limitations.

## 3. Materials and Methods

**Figure 2:**
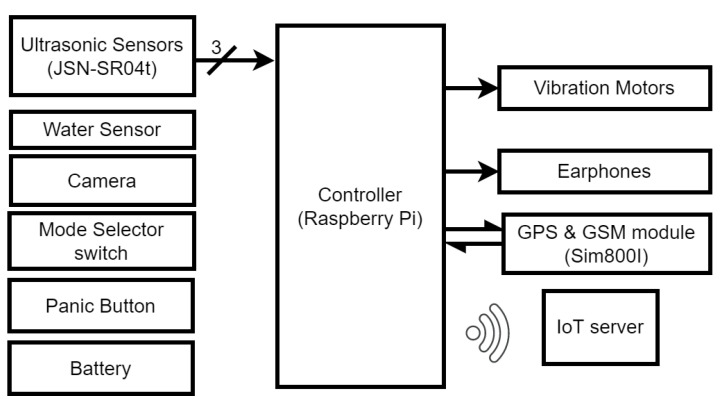
Block diagram of electronic circuitry for the smart stick.

The standard stick traditionally used by visually impaired individuals is replaced by a new, IoT-enabled intelligent stick with obstacle recognition that uses the latest technological advancements, is easy to use, and is packed with features without compromising on battery life, size, or weight. The system offers two modes to detect obstacles and recognize the obstacles in the route of individuals as well as the detection of damp surfaces that should be avoided for safety. The system also offers IoT-based live location sharing on the cloud server, which can be accessed by the user’s caretaker on their smartphone or personal computer by entering their credentials on the server. The credentials are unique for each concerned individual to maintain the privacy of the visually impaired user, and only they can share the credentials with whom they feel comfortable. Figure 2 shows the block diagram of the proposed smart stick.

### 3.1. Physical Design and Criteria

Before discussing the system, the design of the smart stick is mandatory due to the comfort of the user being the top priority. The physical design of the smart stick is based on the following criteria:

- Good hand grip,
- Adjustable for varying heights of users,
- Lightweight.

**Figure 3:**
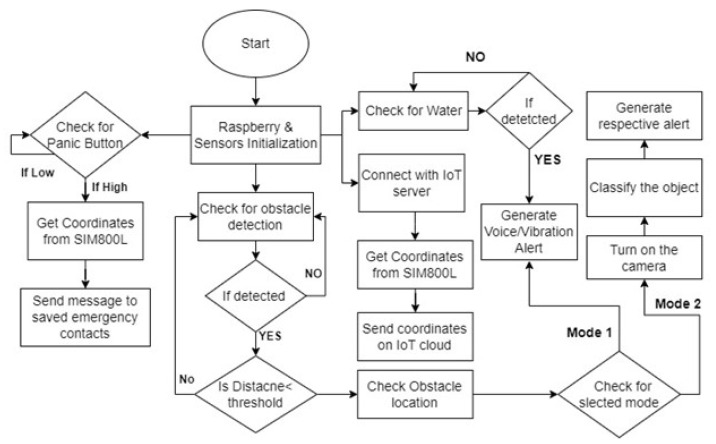
Functional flow diagram of the intelligent stick.

As comfort is the top priority, so the length of the smart stick should be of soft materials, such that the user feels comfortable with it. Generally, the smart stick is the height of the average user’s chest, but it is not suitable for every individual. For this reason, the proposed smart stick is adjustable according to the person’s height. The most comfortable height of the stick is half of the average person’s height, which is 0.86 m. The smart stick can be adjusted accordingly if the person is above or below the average height. Figure 3 shows the workflow of the proposed design.

### 3.2. Material Selection

Material selection is an important step in the fabrication phase. The durability and light weightiness of the system greatly depends on the material. As per our budget constraint, we needed to select a material that should be cheap while being lightweight and durable. For this purpose, we are using synthetic plastic for the handle and aluminum for the rod of the stick due to their low price, durability, availability, and low weight. The handle of the smart stick was also coated with anti-skid sweat-absorbent silicone. Silicone is also known for its wound healing, moisture-locking, and skin-smoothing properties [19]. The following table shows the comparison of the physical properties of the available competitive materials.

From Table 1, it is apparent that stainless steel is stronger than aluminum, but that comes with increased weight and cost. Aluminum is more suitable, however, according to our selection criteria.

### 3.3. CAD Design

**Figure 4:**
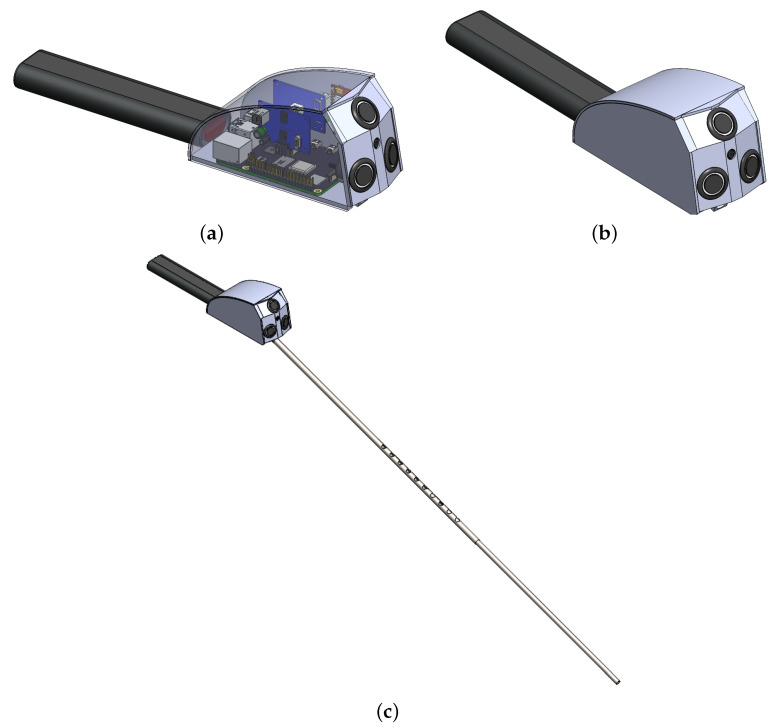
(**a**) Electronic circuitry placement; (**b**) Control box design; (**c**) Design of the adjustable IoT-enabled Intelligent Stick.

On the top of the stick, there is a power ON/OFF switch and a function (mode) selection switch for choosing between obstacle detection only through ultrasonic sensors for longer battery life or obstacle detection and recognition using image processing for more accessibility. The functional flow diagram to switch between different modes is provided in Figure 3. Lastly, a panic button is placed for sending the location of the user to their caretaker through GPS/GSM. The CAD design is shown in Figure 4.

### 3.4. Sensors’ Placement

**Figure 5:**
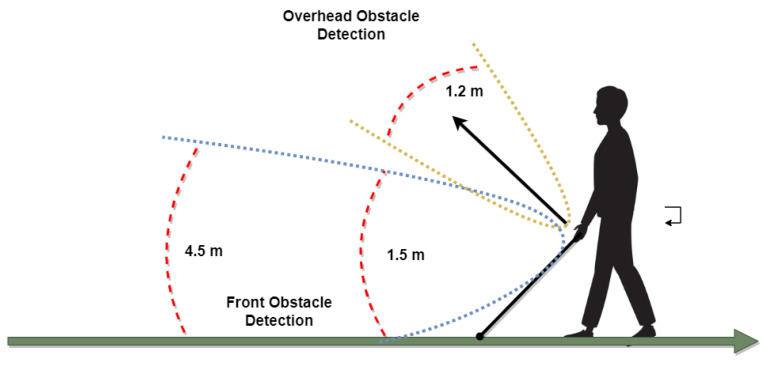
Ultrasonic sensors’ placement on the smart stick.

The sensors’ placement is shown in Figure 5; each sensor has been placed at different angles for maximum coverage of the area in front and allowing for different obstacle detection. The maximum detecting range is 3.5 m; however, the maximum allowable range by the ultrasonic sensor is 4.5 m. Overhead obstacles are detected up to 1.2 m, since only these obstacles need to be avoided by the user.

### 3.5. Obstacle Detection and Recognition

Obstacle detection is the main aim of this smart stick, and for that, ultrasonic sensors are used. An ultrasonic sensor detects any object that comes across the way of the user using its high-frequency waves. Those ultrasound waves are reflected back by the object and are detected by the ultrasound sensor. By measuring how much time has elapsed between transmitting and receiving sound waves, the distance between the sensor and the object can be measured.

(1) $$Distance\left(\mathrm{cm}\right)=\frac{\mathrm{Speed}\mathrm{of}\mathrm{sound}(\mathrm{cm}/\mu s)\times Time(\mu s)}{2}$$

If the object detected is within a specified distance, the controller will generate an alert for the obstacle and the relevant audio message is played, and also the relevant vibration motor is turned on. The obstacle detection has been conducted using both ultrasonic sensors and the Raspberry Pi camera. The obstacle recognition is conducted using the OpenCV and Raspberry Pi camera. Thus, the smart stick has two modes.

#### 3.5.1. First Mode with Detection Only

**Figure 6:**
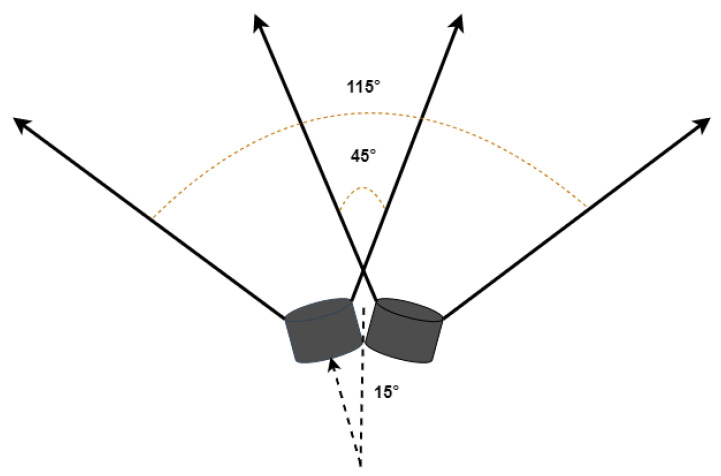
Ultrasonic sensors’ placement.

Three ultrasonic sensors are used for obstacle detection. Two are mounted in front at an angle of 15° from the front. The ultrasonic sensors used are jsn sr04t waterproof ultrasonic sensors with a field of view (FOV) of 75°. Thus, with a specific arrangement, the maximum area is covered in front. A 45° using both sensors is considered as the front, and the total FOV is 115°, as shown in Figure 6. Any obstacle detected by the left sensor only plays the audio message for the left obstacle and turns on the right vibration motor to guide the visually impaired person to the right and vice versa for the right obstacle. When both the sensors detect an obstacle, the obstacle is in front, and the front audio message is played, but no vibration motors are turned on, indicating that the user can move in any direction. The vibration intensity is dependent on the distance between the obstacle and the user. The intensity increases as the user move closer to the obstacle.

Another ultrasonic sensor has been mounted on the top to allow for overhead obstacle detection, such that the user does not bump their head onto it. This sensor is placed at a 45° angle to detect incoming overhead obstacles. The audio for the overhead obstacle is played whenever one is detected. The range for this sensor to detect an obstacle has been limited to 1.2 m so that it does not detect overhead obstacles that can easily be passed under by the user without ducking.

#### 3.5.2. Second Mode with Detection and Recognition

The second mode consists of utilizing image processing to detect the obstacles and recognize them using the OpenCV library. The algorithm for detecting the obstacles detects the common objects found on the street, e.g., it can differentiate between a human, stairs, car, motorcycle, chair, table, trashcan, pit, and signpost. An audio message for the type of object recognized is played through the earphones to avoid noise pollution. Earphones are directly connected to the Raspberry audio jack; e.g., if a car is detected, the audio message that says ‘car detected’ is played. OpenCV is used to program real-time computer vision, and the algorithm for obstacle detection was inspired by the work of the [20].

### 3.6. Water Detection

**Figure 7:**
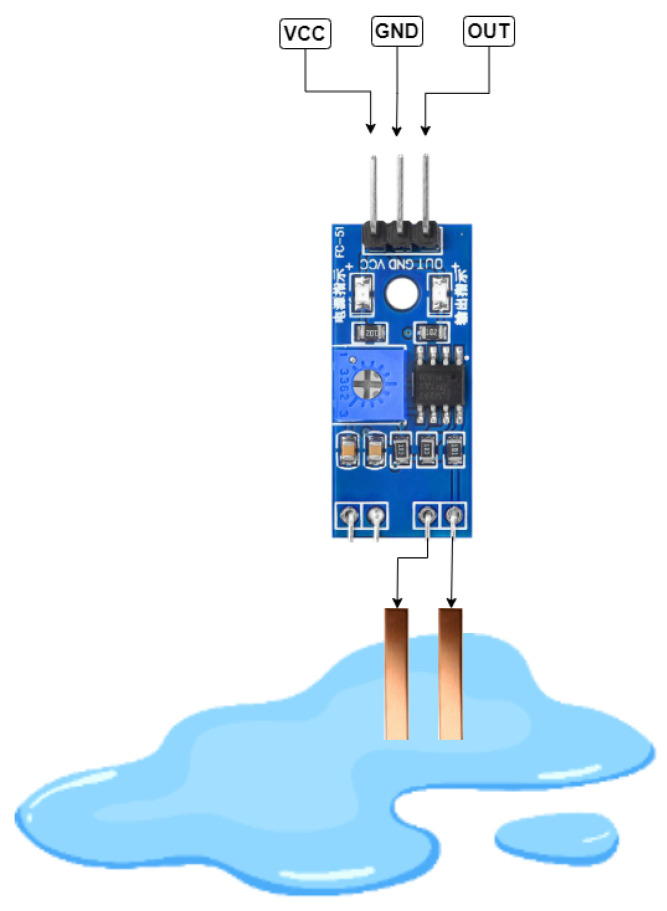
Water detection using copper terminals.

For water detection, the copper shorting technique has been used. For this purpose, a custom water sensor was designed using two copper terminals that were made from copper sheets and connected to the receiver terminal of the IR sensor. When water comes across the path, copper terminals get in contact with it, and water, being an excellent conductor, conducts and causes the short-circuiting resulting output pin of the sensor to go HIGH and send a signal to the controller, which then generates an audio alert for water detection. The water detection mechanism is shown in Figure 7.

### 3.7. GPS/GSM Module

**Figure 8:**
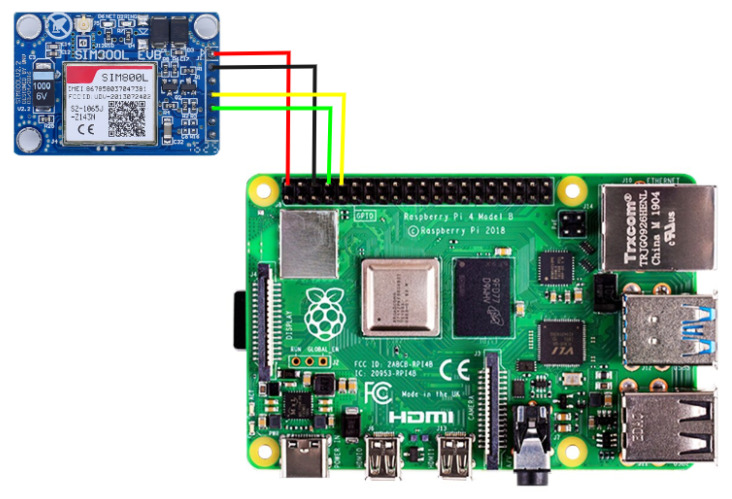
GSM with controller for sending location.

When the user misplaces the smart stick and needs any assistance in finding it, a trigger button on the stick has been implemented to send their location to their specified caretakers via SMS. For this purpose, SIM800l is used, which is a low-cost and small-size GSM/GPS module that can be integrated into different IoT projects. The GSM controller is shown in Figure 8. This module was selected due to its low operating voltage, which ranges from 3.4 V to 4.4 V. It makes it a perfect candidate for our application, achieving a longer battery life.

### 3.8. IoT-Based Location Sharing

Using the previously mentioned GPS, the live location of the user is measured and sent to the IoT server every 10 s using the GSM that is connected to the internet. This allows the caretakers to keep track of the visually impaired individual or track the location of the stick if misplaced. Pressing the panic button and sending a location in an SMS through GPS/GSM is an emergency alert to the caretaker, while location sharing through an IoT server is for finding the lost stick or the person globally, unlike the RF modules that have been generally used in literature that only work locally.

### 3.9. Experimental Setup

**Figure 9:**
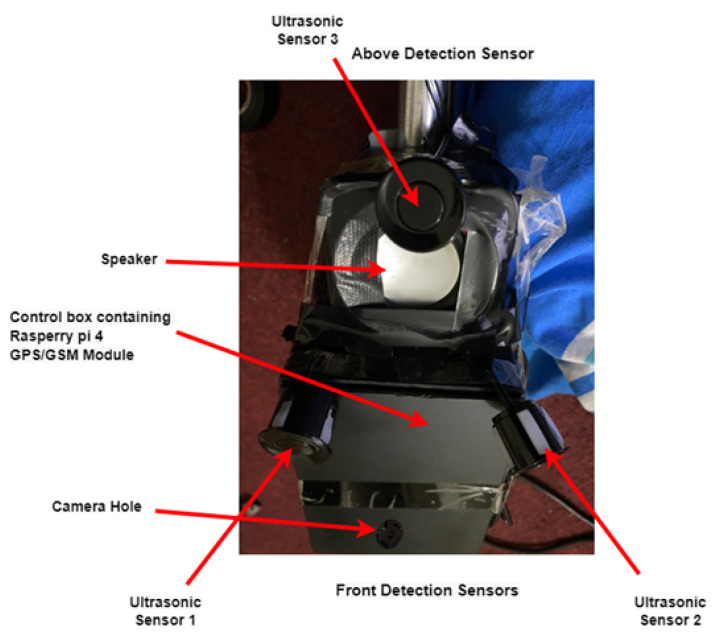
Experimental setup of the intelligent stick for the visually impaired.

The following Figure 9 shows the experimental setup along with all sensors of the smart stick for the visually impaired. The water sensor was deployed at the bottom of the stick.

The following Table 2 shows the Instruction Set Architecture (ISA) and Central Processing Unit (CPU) architecture along with other specifications of the Raspberry Pi 4 used in our stick.

Although Raspberry Pi 4 is a more portable, efficient, low cost and lower-power device than the available microcontrollers such as jetson nano, Asus Tinker Board S R2.0, etc., it has been further optimized for low power consumption by disabling the following, as they are not utilized in our stick:

- Disabling HDMI output,
- Disabling the USB controller,
- Disabling Wi-Fi and Bluetooth,
- Disabling onboard LEDs.

**Figure 10:**
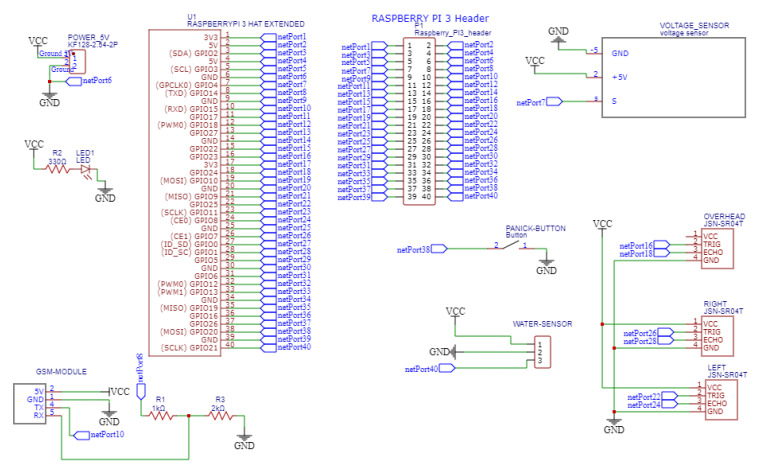
Schematic diagram of the proposed design.

Figure 10 shows the schematic diagram of the proposed design. For the water sensor, the GPIO port is pulled up to avoid false detection and a falling edge interrupt was used for calling the interrupt service routine (ISR). The same falling edge interrupt was used for ultrasonic sensors and their GPIO ports are pulled up as well.

## 4. Results

A usable product for visually impaired persons in the form of a smart stick is designed. The two ultrasonic sensors detect the obstacles at a maximum range of 4.5 m and a minimum range of 0.25 m. The ultrasonic sensor detects the obstacles successfully at a range of 3.5 m, after which the output was uncertain. The final detection range is set to 3.5 m, and if any of the obstacles are out of the range, they would not be considered obstacles until they came into the specified range, as shown in Table 3.

**Figure 11:**
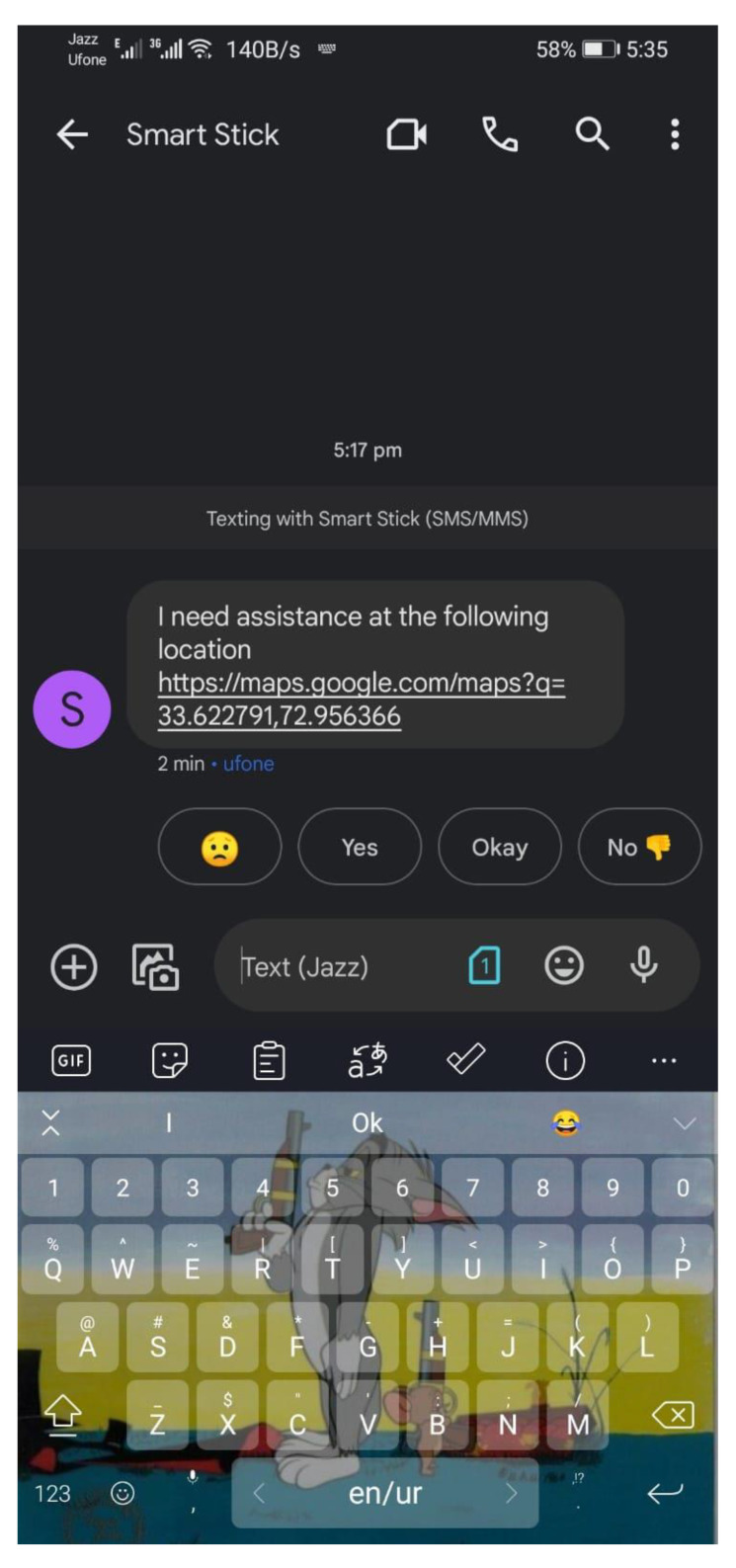
Location sharing SMS through GSM.

The intensity of the vibration motors varies with the distance from the obstacle. The pulse width modulation (PWM) of the motor is increased, as the distance from the obstacle is decreased. Thus, the vibrations become more and more intense, indicating the closeness of the obstacle to the user. Thus, haptic feedback plays an important role in the smart stick. The water sensor is mounted 5 mm above the bottom edge of the stick so that it only senses water that is actually in the form of a puddle and dangerous for the user. In the testing phase, it worked as intended and fulfilled its purpose of detecting water puddles. SIM8001 was used to obtain the latitude and longitude values of the user’s location through GPS, and the message was sent to the caretaker’s mobile number using the GSM. Figure 11 shows the message sent to the caretaker automatically after the panic button is pressed on the handle of the smart stick. The location is sent as a Google Maps link of the format http://maps.google.com/maps?q=loc: with the latitude and longitude values at the end separated by commas, which can then be opened by the caretaker. The message “I need assistance at the following location” is also sent before the link to Google Maps. Different phone numbers can be added as per requirement.

**Figure 12:**
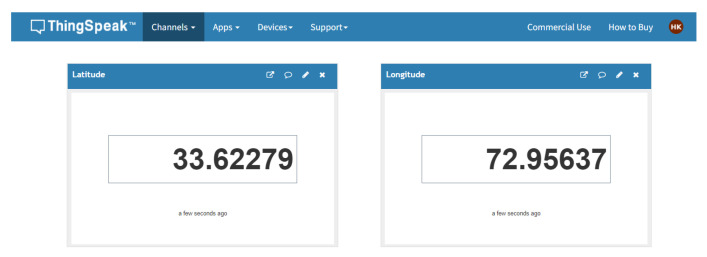
ThingSpeak IoT platform location sharing.

The ThingSpeak IoT platform is utilized as the IoT server for location sharing. The location sending interval has been set to 10 seconds to avoid overloading the server and maintaining a record of the locations visited by the user on the server. The location is captured in terms of latitude and longitude using the GPS and sent through the GSM of SIM8001, as shown in Figure 12, which is connected to the IoT platform. If the internet is not available at any location, the location sharing through the IoT platform will be disabled until the connection is restored. The disabling of the IoT platform does not affect the rest of the system.

**Figure 13:**
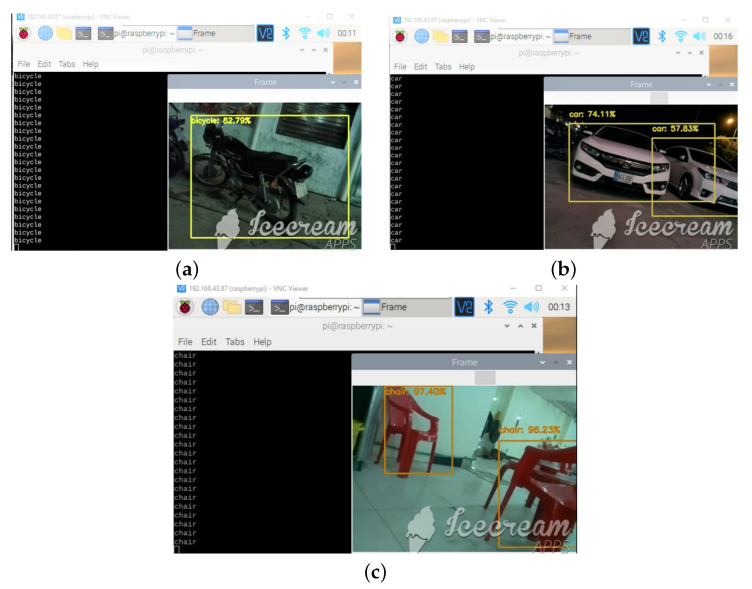
(**a**) Motorcycle detected using OpenCV; (**b**) Cars detected using OpenCV; (**c**) Chairs detected using OpenCV.

OpenCV is used as the image processing library to detect and recognize the objects in the scene captured using a Raspberry pi camera and processed on the Raspberry pi 4. Figure 13a–c shows the bicycle, cars, and chairs detected by the algorithm. After the object is recognized, the earphones play an audio message about the type of object recognized, e.g., if a car is detected, "car detected" is played on the earphones. The function (mode) selection switch was also successfully used to change between the first and second modes, discussed previously. The dataset used for training the OpenCV algorithm for object detection and recognition using the Single Shot MultiBox Detector (SSDs) method is publicly available for use at: https://pyimagesearch.com/2017/09/11/object-detection-with-deep-learning-and-opencv/ (accessed on 20 April 2022). The camera gave 8 frames per second (FPS), which were utilized for object detection and recognition by the algorithm. Other real-time constraints include a specific number of objects that are recognizable, and various obstacles such as humans, stairs, cars, motorcycles, chairs, tables, trashcans, pits, and signposts. Moving vehicles with speeds greater than 25 mph are not efficiently recognized by the algorithm.

Table 4 shows the comparison of different types of smart sticks found in the literature with the proposed IoT-enabled intelligent stick for the visually impaired. Along with the mentioned features, the proposed stick for the visually impaired also contains overhead obstacle detection. It also has the global stick-finding capability and live location on the IoT dashboard enabled using the ThingSpeak IoT platform. The measuring range of the overhead obstacles has been adjusted through experimentation to a value of 1.2 m so that only the objects that can come in contact with the user are considered overhead obstacles. Although obstacle recognition has been implemented in various fields, very few smart sticks used it. The proposed smart stick is the first successful implementation that utilizes this feature along with the standard obstacle detection features. Various obstacles, such as humans, stairs, cars, motorcycles, chairs, tables, trashcans, pits, and signposts, that are commonly found in the life of a visually impaired person, are recognized by the proposed system. Thus, as per the literature review, no other smart stick had all these mentioned features combined to create a smart stick that helps the visually impaired in their daily lives.

This implementation of the proposed intelligent stick is tested by three people with different visual constraints:

1. Visually impaired person;
2. Blind person;
3. Person whose vision was purposely impaired with a face mask to mimic newly blinded people.

**Figure 14:**
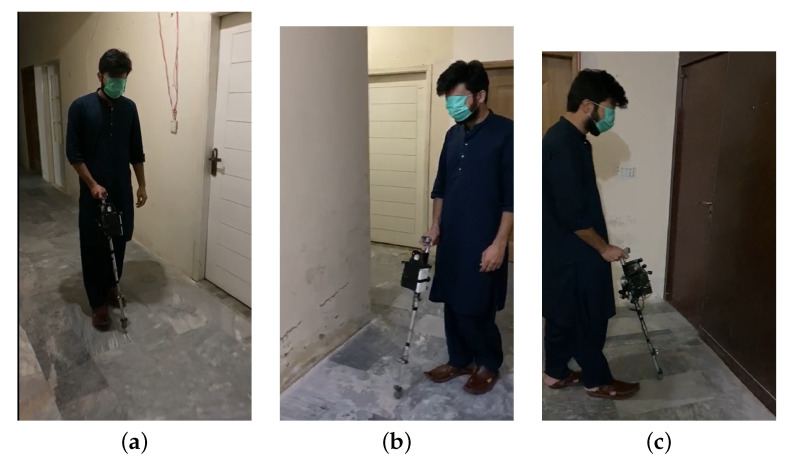
(**a**) Lab testing of the stick; (**b**) Side Obstacle Detected; (**c**) Front Obstacle Detected.

The feedback from all three is reassuring and the simplicity of the smart stick and the very low learning curve were the highlights of their feedback. Their feedback demonstrated that this implementation of the intelligent stick with the such simple technology of ultrasonic sensors, water sensors, and some complex technologies of machine learning through the camera, and GPS/GSM location sharing through IoT has helped them in roaming the environment easier than with a traditional cane that they were using before. The additional experiments that have been conducted to highlight the features and limitations of the smart stick are:

1. IoT application in the countryside, where the internet is not available, demonstrated that the smart stick worked successfully by disabling the location sharing on the IoT platform and not causing any interruptions whatsoever.
2. Experimentation in overcrowded areas demonstrated the limitation of our design, where the sensors had no distance to work with and thus, gave high readings most of the time. The second limitation was faced by the camera, where the images were captured for detection and the recognition was not clear enough in the crowded area for any detection or recognition. Moving vehicles with a speed greater than approximately 30 km/h are not efficiently recognized by the algorithm as well.
3. Indoor testing of the smart disk is shown in Figure 14. The face mask was used to cover the eyes of the user, as stated above.

### Average Power Consumption

The following Table 5 shows the current drawn by each component of the device. Mode 1 using ultrasonic sensors draws less current than Mode 2 using the Raspberry pi camera.

A 5V/2.1A 5000 mAh Lithium-ion battery has been used for powering the device. The battery is cylindrical in shape because it has to fit in the cylindrical handle of the stick. The following Table 6 shows the total power consumption in Mode 1 and Mode 2 along with the total battery timing in both modes.

In Table 6, the total current drawn is simply a sum of the current drawn by different components in each mode. Mode 1 uses ultrasonic sensors, while Mode 2 uses the Raspberry pi camera. Average power consumption can be found using P = VI, and battery timing is calculated by dividing the 5000 mAh rating of the battery by the total current drawn.

## 5. Discussion

The design of the stick is well thought out for each heightened person and, thus, is made adjustable for every person’s use. The stick is made of aluminum for the minimum weight possible in the budget, but the different available materials have been compared in Table 1. Further research on different polymers, metals, and composites can be conducted to optimize the weight and strength ratio of the stick. Furthermore, the stick is capable of detecting an obstacle and informing the user through audio and/or haptic feedback before they come in contact with it to avoid accidentally knocking into an object and hurting themselves. The stick can detect water if it comes in front of the user and warns them through an audio message, thus avoiding any mishaps or wetting. One of the most notable features of the IoT-enabled intelligent stick is that it can detect obstacles and with the help of image processing using OpenCV, it can identify obstacle types as well. Moreover, it can share the location of the user globally through the IoT platform to the caretakers or the location of the stick to the IoT platform, if it gets lost. Different obstacles that can be differentiated are humans, stairs, cars, motorcycles, chairs, tables, trashcans, pits, and signposts. If the user is lost or is in trouble and needs assistance, he/she can send an SMS containing the Google Maps link containing the location of the user to the caretaker by triggering the panic button mounted on the handle of the smart stick. The feedback from the vibration motors and audio feedback helps the user to navigate their environment easily, which is evident from the initial testing discussed in Section 4.

From the above discussion, the features have been highlighted, but there are some limitations to the device discussed in Section 4. Such limitations reduce the usefulness of the device in some cases where they occur. One of the limitations, that it is overcrowded and navigation cannot be conducted with the use of the device, can be navigated by the user through their natural senses of touch and hearing and using the device as a traditional cane; the stick can provide audio directions on where to go using Google Maps. This can be one of the ways to solve this limitation to an extent in a future study. Although the testing by people having various visual constraints approved of the device, in the future, however, a survey could be conducted to provide statistical results that infer the usefulness of the device to potential users. Furthermore, different future steps discussed in Section 6 can be implemented and tested to compare the statistical results and determine whether the improvement drastically improved the potential user’s reviews.

## 6. Conclusions and Future Steps

Visually impaired people need assistance to navigate safely for daily activities, and often a dog or stick is used for that purpose. With the advancements in technology, assistive sensors have been introduced in modern sticks for object detection and to guide the users to navigate on the outside. This study presents a smart stick that can both detect and identify obstacles and help visually impaired people. The blind population may go out into the world without any help from others while utilizing the proposed IoT-enabled intelligent stick with obstacle recognition because of its various features, including obstacle detection and recognition, water puddle detection, audio messages, haptic feedback, live location sharing and panic button for emergency contact through SMS. Furthermore, the proposed smart stick is fully automated and does not need any input from the user, as it consists of ultrasonic sensors and camera modules that sense obstacles around the user and provide feedback to the user with the assistance of earphones and vibration motors connected to the controller. The two modes of obstacle detection and obstacle detection and recognition are useful in their own ways; the first has a longer battery life, but the latter has more accessibility features. The system continually operates so that the visually disabled person can obtain updates on the challenges up ahead at any point in the way. Consequently, with the aid of this intelligent stick, the visually impaired can perform their daily tasks comfortably and go out in the world without any fear of getting lost or hitting someone or something.

Although the proposed design comes with various features, as nothing is perfect, it still requires some improvements that can be conducted in a future study, such as:

- Allowing for global live tracking without the use of an IoT platform.
- Enabling the detection and recognition of objects in an overcrowded environment.
- The use of a high-quality camera while maintaining low power consumption for better image quality for detection and recognition.
- Further optimization of the battery consumption by using the latest microelectromechanical systems (MEMS) based sensors that are known for their low power consumption, while maintaining the performance parameters.
- Updating the various types of obstacles that can be detected and recognized other than those discussed in Section 3.5.2 “Second Mode with Detection and Recognition”.
- Detection and recognition of vehicles with speed greater than 30 km/h.
- Further research on different polymers, metals, and composites can be conducted to optimize the weight-to-strength ratio of the stick.
- Conducting a survey on the fabricated stick’s perceived usefulness for potential users and providing statistical results, as such statistical results are beyond the scope of this manuscript.

Therefore, an intelligent stick was designed and tested for the visually impaired having various features and room for improvement in the future using the latest technologies. 

## Figures and Tables

**Table 1:** Comparison of available materials for stick.

Physical Parameters	Aluminum 6061-T6	Stainless Steel 316
Yield strength	155 MPa	205 MPa
Density	2.7 [g/cm^3^]	8 [g/cm^3^]
Elastic modulus	68.9 GPa	193 GPa
Elongation (% in 50 mm)	22	40

**Table 2:** Raspberry pi specifications.

Processor	Broadcom BCM2711, Quad-Core Cortex-A72 (ARM v8 ISA) 64-bit SoC @ 1.5 GHz
RAM	4GB LPDDR4-3200 SDRAM
Clock Speed	1.5 GHz
GPIO	Standard 40-pin GPIO Header
Operating System	Raspberry Pi OS

**Table 3:** Actual and measured distance.

Actual Distance	Output	Measured Distance
0.25 m	High	0.261 m
1.5 m	High	1.489 m
3.5 m	High	3.477 m
4.5 m	Low	0 m

**Table 4:** Comparison with existing literature.

Ref.	Sensors	Output	Controller	GPS/GSM	IoT Enabled?	Image Processing?	Detection Range (Max)	Overhead Detection?	Water-Proof?
[13]	Ultrasonic sensor, Light sensor	LEDs, Buzzer	Arduino	No	No	No	15 cm	No	No
[16]	Ultrasonic sensors, moisture sensor, RF module	Buzzers, vibration motors	Arduino	No	No	No	70 cm	No	No
[15]	Ultrasonic sensors, IR sensor, water sensor	SD card module, speaker	Arduino	No	No	No	Not specified	No	No
[11]	Ultrasonic sensors, moisture sensor, IR sensor, RF module	Speaker, buzzer, vibration motor	Arduino	Yes	No	No	450 cm	No	No
[10]	Ultrasonic sensor	Buzzer, vibration motor, speaker	Raspberry Pi	Yes	No	No	300 cm	No	No
[5]	Ultrasonic sensor, moisture sensor, RF module	LCD, buzzer	Arduino	No	No	No	200 cm	No	No
[9]	Ultrasonic sensor, water sensor, RF module	Vibration motor, buzzer	MSP430	Yes	No	No	Not specified	No	No
Proposed	Ultrasonic sensors, water sensor	Speakers, vibration motors	Raspberry Pi	Yes	Yes	Yes	350 cm	Yes	Yes

**Table 5:** Current drawn by different components.

Component	Average Current Draw
Ultrasonic Sensors (×3)	90 mA
GPS/GSM Module	300 mA
Water Sensor	20 mA
Raspberry Pi 4 (Mode 1)	700 mA
Raspberry Pi 4 (Mode 2)	1300 mA

**Table 6:** Average power consumption and battery timing.

Mode	Total Current Drawn	Average Power Consumption	Battery Timing
1	1110 mA	5.55 W	4 h 30 min
2	1620 mA	8.1 W	3 h 5 min

## Data Availability

Not applicable.
